# A Subphase-Labeled Mitotic Dataset for AI-powered Cell Division Analysis

**DOI:** 10.1038/s41597-026-07007-7

**Published:** 2026-03-13

**Authors:** Zsanett Zsofia Ivan, Dominik Hirling, Istvan Grexa, Jonas Ammeling, Csaba Molnar, Tamas Micsik, Katalin Dobra, Levente Kuthi, Farkas Sukosd, Janos Fillinger, Judit Moldvay, Erika Toth, Marc Aubreville, Vivien Miczan, Peter Horvath

**Affiliations:** 1 HUN-REN Biological Research Centre, Szeged, Hungary; 2 Single-Cell Technologies Ltd., Szeged, Hungary; 3 Doctoral School of Biology, University of Szeged, Szeged, Hungary; 4 Technische Hochschule Ingolstadt, Ingolstadt, Germany; 5 Karolinska Institutet, Department of Oncology-Pathology, Solna, Sweden; 6 Department of Clinical Pathology and Cytology, Karolinska University Hospital, 171 77 Solna, Sweden; 7 Department of Surgical and Molecular Pathology, Tumor Pathology Center, National Institute of Oncology, Budapest, Hungary; 8 Department of Pathology and Experimental Cancer Research, Semmelweis University, Budapest, Hungary; 9 HUN-REN-ONKOL-TTK-HCEMM Oncogenomics Research Group, National Institute of Oncology, Budapest, Hungary; 10 Department of Pathology, Péterfy Sándor Street Hospital and Outpatient Clinic, Budapest, Hungary; 11 1st Department of Pulmonology, National Koranyi Institute of Pulmonology, Budapest, Hungary; 12 Department of Pulmonology, University of Szeged Albert Szent-Gyorgyi Medical School, Szeged, Hungary; 13 Translational Oncopulmonology Research Group, Institute of Molecular Life Sciences, HUN-REN Research Centre for Natural Sciences, Budapest, Hungary; 14 Flensburg University of Applied Sciences, Flensburg, Germany; 15 Institute of AI for Health, Helmholtz Zentrum München, Neuherberg, Germany; 16 Institute for Molecular Medicine Finland, University of Helsinki, Helsinki, Finland

**Keywords:** Computational models, Data publication and archiving, Cancer imaging, Mitosis

## Abstract

Mitosis detection represents a critical task in digital pathology, as it plays an important role in the tumor grading and prognosis of patients. Manual determination is a labor-intensive task for practitioners with high interobserver variability, thus, automation is a priority. There has been substantial progress towards creating robust mitosis detection algorithms, primarily driven by the Mitosis Domain Generalization (MIDOG) challenges. Also, there has been growing interest in the molecular characterization of mitosis to achieve a more comprehensive understanding of its underlying mechanisms in a subphase-specific manner. We introduce a new mitotic figure dataset annotated with subphase information based on the MIDOG++ dataset as well as a previously unrepresented tumor domain to enhance the diversity and applicability. We envision a new perspective for domain generalization by improving model performance with subtyping mitosis, complemented with an atypical mitotic class. Our work has implications in two main areas: subtyping information can provide helpful information in mitosis detection, while also providing promising new directions in answering biological questions, such as molecular analysis of subphases.

## Background & Summary

The field of digital pathology has undergone tremendous progress in recent years, for which the appearance of large vision models and the convergence of methods in the computer vision field have paved the way. Mitosis detection takes up a fairly small portion of the whole discipline, however, it has become an important area for research mainly due to its clinical relevance and the appearance of large, high quality datasets. Consequently, mitotic figure (MF) detection has become a benchmark task for domain robustness as well. The MITOS2012 challenge^1^ introduced the first publicly available mitotic figure dataset. Here, domain robustness was not a priority, as the training and test sets were from the same histology slides. Subsequent challenges, such as MITOS2014^2^, AMIDA13^3^, and TUPAC16^4^, used breast cancer data and included more cases, but were limited by using only two scanners for the training and test sets. The MIDOG (MItosis DOmain Generalization) challenges were created to promote the development of domain robust detection algorithms. The MIDOG 2021 challenge^5^ used a training set of 200 cases from four scanning systems and a test set of 100 cases from four scanning systems, including two previously unseen scanners. The MIDOG 2022^5,6^ challenge included considerable diversity of tissue types and species. MIDOG++^7^ extended these datasets to include 2 mm^2^ regions of interest from 503 WSIs of seven different tumor types and labels for 11,937 mitotic figures.

Network architecture extensions with gradient reversal layers^8,9^, domain generalization capabilities of networks^10,11^, novel domain augmentation techniques^12–14^ and a proper selection of unsupervised pre-training tasks^11^ have all seen substantial benefit from the diverse data provided by mitosis detection research. State-of-the-art approaches exclusively use deep learning (either convolutional neural networks or vision transformers) to address the challenges of mitosis detection. Most of the published methods are framing the task as an object detection challenge, but sliding-window approaches have also been successfully applied at the cost of a higher running time^15^. The state-of-the-art MF detection method takes inspiration from the field of behavioral psychology and aims to replicate the multi-scale reasoning typical of pathologists: a “macro-vision” step is performed which yields the semantic segmentation of mitotic candidates and a second “micro-vision” step performs the filtering of imposter cells^11^.

A significant challenge in applying mitosis detection algorithms to real-world diagnostic processes is the drop in performance when models are tested on data from different domains. Domain shifts can result from differences in species, organ type, scanner variability, or staining protocols. To address this, tissue-specific data augmentation techniques, such as stain augmentation and stain normalization, have been used to improve the generalization capabilities of mitosis detection algorithms^11,12^. Stain augmentation can be achieved using classical methods by estimating the parameters of the staining and the scanner, or by using frequency-based approaches, such as Fourier Domain Adaptation (FDA), which swaps the low-frequency spectrum of source and target images to perform a stain adaptation between different scanners^13,14^. Besides staining-related augmentation techniques, modifications to network architectures also proved to be an effective way to achieve domain generalization: the reference algorithm for the MIDOG2021 challenge employed a domain-adversarial training technique, which involves adding a domain classification branch to the network to encourage domain-invariant features^9^.

The assessment of atypical mitotic figures (AMFs) has also gained attention as a potential prognostic marker in breast cancer. The AMi-Br^16^ is one of the first datasets to include subtype information related to mitotic figures. A hierarchical anchor-free detection method has been developed to solve the mitotic vs. AMF subtyping problem^17^ and the results imply that performing the classification of mitotic cells helps to improve the performance of the original MF detection task. To our knowledge, there is not yet any publicly available dataset or study that tackles the question of what happens if the mitotic cells are subtyped into the 5 different stages of normal mitosis (pro-, prometa-, meta-, ana-, and telophase) with atypical mitotic figures as a separate class.

Despite all the advancements in the research of mitosis, current methods often treat mitotic figures as a single category, overlooking potential subtypes that may hold clinical significance such as the number of atypical MFs, that have been associated with adverse prognostic factors, including reduced overall survival and poorer clinical outcomes^16,18,19^. In addition to this, the classification of mitosis can open up further relevant questions in research, in particular when complemented by molecular analysis^20–22^. Recent studies suggest that subtyping mitotic figures could lead to better model generalization and improved interpretability^17^, yet publicly available datasets incorporating such annotations remain scarce. Moreover, while modern network architectures and augmentation strategies have pushed detection performance forward, there remains room for improvement in both object detection and classification strategies, particularly in capturing the ordinal relationships between different mitotic phases.

**Fig. 1:**
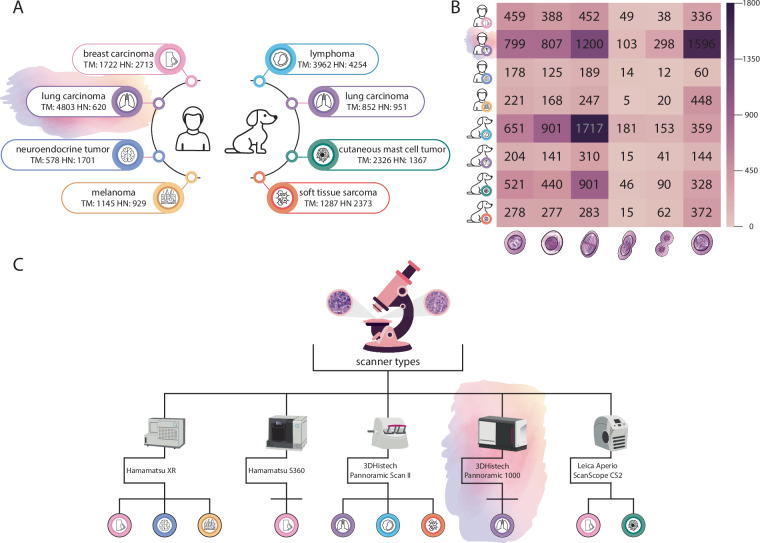
A comprehensive summary of data across species, tissues, tumor types, microscopes and mitotic figure numbers, highlighting our contribution. (**A**) Overview of available mitotic datasets categorized by species, tumor type, and tissue of origin, complemented by the total mitotic (TM) and hard negative (HN) figure numbers (**B**) Comparative heatmap with mitotic figure number distribution (normal prophase, prometaphase, metaphase, anaphase, telophase, and atypical mitosis, respectively) of the different tumor types (**C**) Summary of scanner models and the corresponding tissue specimens imaged using each device.

To address these problems, we propose a new approach that pushes existing mitosis detection workflows forward in two key areas:

- We create an annotated dataset extending MIDOG++ with detailed subtyping information. In addition to bounding boxes, we provide corresponding segmentation masks to achieve more precise localization. The necessity of segmentation is on one hand supported by the potential need for morphological analysis of the dividing cells for grading purposes. Another use-case that we envision is the isolation and molecular analysis of the mitotic cells via laser microdissection, for which accurate cell boundaries are necessary^23^. Furthermore, we release an additional benchmarking dataset created from human lung adenocarcinoma (named LUNG-MITO), since it is the most common histological subtype of lung cancer with a heavy mortality burden. We publicly release the whole dataset for research purposes (Fig. 1).
- We develop a novel mitosis detection and segmentation pipeline. Our segmentation framework is built upon Mask R-CNN, a region proposal-based approach. We replace the traditional ResNet-50 backbone with ConvNeXt, a state-of-the-art feature extractor and apply a hierarchical candidate refinement step. Our pipeline also incorporates domain augmentation strategies during training.

## Methods

### Specimen preparation and digitalization

For the LUNG-MITO dataset^24^ (approved under ethical license numbers BM/22651-1/2024 and BM/27289-1/2025 issued by the Scientific and Research Ethics Committee of the Medical Research Council -ETT TUKEB) 21 whole slides from 10 lung adenocarcinoma patients were selected where the tumor region was prominent. The experiments conducted in this study did not involve any additional procedures or interventions for the patients. All tissue samples were obtained as part of the routine diagnostic workflow, and the corresponding pathology blocks were stored in a biobank in an anonymized form. All resected tumor samples underwent routine diagnostic evaluation either at the Department of Pathology, Faculty of General Medicine, Szent-Györgyi Albert University of Szeged or at the 1st Department of Pulmonology, National Koranyi Institute of Pulmonology, Budapest. From archived formalin-fixed paraffin-embedded tissue blocks, 5 μm sections were prepared and hematoxylin-eosin staining was performed. All samples were prepared using routine pathology protocols (see Supplementary Information document Table S1). All cases were scanned with 3DHISTECH PANNORAMIC 1000 (0.24 μm/pixel) and the standard settings were used with 40× magnification.

### Expert labeled dataset description

#### LUNG-MITO dataset^24^

**Fig. 2:**
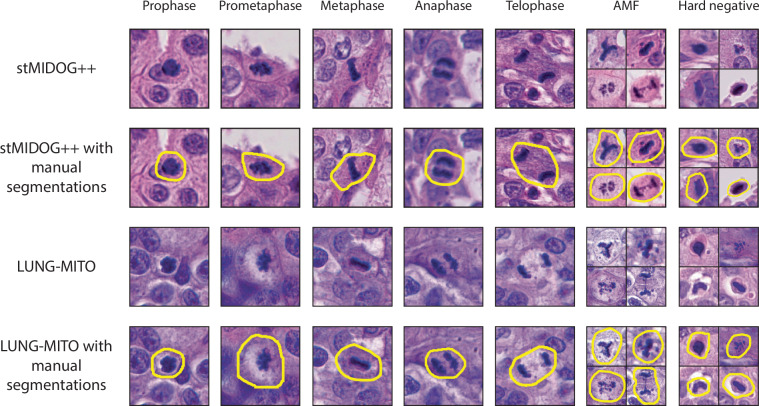
Representative images from the stMIDOG++ and the new LUNG-MITO datasets with manual segmentations. Column 1–5. Normal mitotic figures. Column 6. AMF (atypical mitotic figures) Column 7. Hard negative examples Magnification: 40× For detailed information also see Supplementary Information document Table S2.

Our mitotic figure dataset consists of human lung adenocarcinoma samples from 10 different patients and 21 slides All slides scanned with 3DHISTECH PANNORAMIC 1000. The images had a resolution of 0.24 µm/px, respectively, and were tiled to 1024×1024 parts. In order to include all potential mitotic figure candidates, the whole tumor area was selected for analysis as a region of interest (ROI) from each whole slide image (WSI), rather than only the hot spot regions that are typically suggested by standard pathology protocols^6,25–28^. Then, mitotic cells were selected, segmented manually and assigned into classes of the 5 major normal mitotic phases (1-Prophase, 2-Prometaphase, 3-Metaphase, 4-Anaphase, 5-Telophase) complemented by an atypical mitotic figure (AMF) class according to the previously established standards^18,25,29^ (also see Supplementary Information document Figure S1 and Supplementary Information document Table S2 for our annotation strategy). Annotations were performed using the AnnotatorJ plugin^30^ within Napari and the EXACT annotation software^31^.To aid the deep learning models, hard negative figures were selected based on their morphological characteristics described in^18,25,29^ (Fig. 2). Annotations were made under the supervision of expert pathologists (K.D., T.M., L.K.).

#### stMIDOG++ dataset^24^

A large-scale annotated mitosis dataset with a total of 503 tumor cases from 10 different tumor domains (breast carcinoma, lung carcinoma, lymphoma, cutaneous mast cell tumor, neuroendocrine tumor, soft tissue sarcoma, melanoma), resolution ranging from 0.23 µm/px to 0.25 µm/px. The original dataset used 2 classes: mitotic cells and so-called hard negative instances, which are morphologically similar to mitotic cells, and a 3 expert consensus was provided, represented by bounding box coordinates. Here, we extend this labeled dataset by assigning a class from five common subphases (1-Prophase, 2-Prometaphase, 3-Metaphase, 4-Anaphase, 5-Telophase) and an atypical mitotic figure class (without further classification) to each previously annotated mitotic figure. This step was performed by a trained expert with more than 4 years of experience in labeling and identifying mitotic figures on tissue sections. Manual classification was carried out in Napari AnnotatorJ^30^ and EXACT^31^ annotation software according to previously defined standards (also see Supplementary Information document Figure S1 and Supplementary Information document Table S2). In addition to subtyping, precise manual segmentations were also drawn for each of the cells (Fig. 2). This allows morphological analysis of the candidates or to proceed with single-cell isolation.

### Model description

**Fig. 3:**
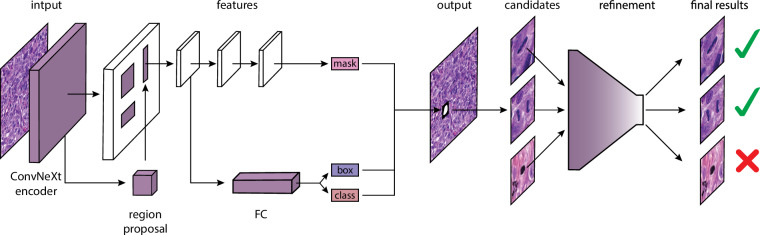
Overview of the proposed two-stage method. First, we propose mitotic candidates with a ConvNeXt encoder-based Mask R-CNN. The candidates are reclassified with EfficientNet in the second step.

Our mitosis detection pipeline (Fig. 3.) consists of 2 major steps: initial segmentation and candidate refinement.

#### Initial segmentation

Our segmentation network is based on Mask R-CNN^32^, a popular deep learning architecture for instance segmentation. Most of the time, traditional backbones such as ResNet-50 or ResNet-101 are used^33^, however, recent advancements in vision transformer-inspired architectures have demonstrated superior feature representation capabilities^34,35^. In our approach, we replace the conventional ResNet-50 backbone with ConvNeXt, a state-of-the-art convolutional neural network that incorporates principles from vision transformers while retaining the efficiency of CNNs^36^. The outputs of our initial method are segmentations and subclasses of mitotic cells.

#### Candidate refinement

To further enhance detection performance, we run an additional classification step on the mitotic candidates yielded by the initial segmentation step. This step is done with EfficientNet, a state-of-the-art classification network^36,37^. The refinement is performed in a hierarchical manner, i.e., first, a mitotic vs. imposter differentiation is performed, and in the second step, the final subphase classification is done on the mitotic cells. An initial decision from Mask R-CNN is only overwritten if its confidence is lower than that of the EfficientNet.

### Evaluation methods

For evaluating the efficiency of our algorithm, the F1 score is calculated in the same manner as in the MIDOG competitions: a segmentation is counted as a true positive (TP), if the centroid of the prediction is matched to a ground truth (GT) segmentation within its pixel radius *r* (for the sake of consistency, we opted for *r=25* similarly to the MIDOG evaluation metric). A ground truth object can only have one corresponding match, every other prediction will be counted as a false positive (FP). Those GT segmentations that don’t have any corresponding matches will be counted as false negative (FN).

Because of the imbalanced number of mitotic cells in the images, we aggregate our results across all images and calculate the final metric in the end:

$$F1\,=\frac{2\cdot {TP}}{2\cdot {TP}+{FP}+{FN}}.$$

Besides a class-agnostic score, we also report class-wise (cw) metrics, where TP, FP and FN samples are yielded based on detection and classification accuracy.

## Data Records

The images of MIDOG++ are publicly available at the MIDOG++ GitHub Repository (https://github.com/DeepMicroscopy/MIDOGpp) under an MIT license which permits the application described in this paper, while the LUNG-MITO dataset, containing 3526 fully anonymized PNG images, is uploaded as a zipped archive to Zenodo^24^ under a CC BY 4.0 license.

The dataset consists of two types of data: images and the corresponding annotations in COCO format^38^. The images are 1024×1024 RGB tiles from HE-stained tumor regions from ten lung adenocarcinoma patients. The COCO annotation files include image metadata, object-level segmentation masks, bounding boxes, and category definitions. In the stMIDOG++ dataset^24^, we preserved the original filenames from MIDOG++. In the LUNG-MITO dataset^24^, the images are anonymized and named with numeric identifiers (e.g., 00002.png). In both COCO files, each image entry includes a unique image_id used to associate annotations with their respective images. We provide two annotation files: *MIDOGpp_subtyping.json* for the stMIDOG++ dataset^24^, containing 26283 annotations for 503 images, and *Lung_mito.json* for the LUNG-MITO dataset, containing 5423 annotations for 3526 images. Each object is labeled with a category ID from 1 to 7, corresponding to the following categories: 1 – prophase, 2 – prometaphase, 3 – metaphase, 4 – anaphase, 5 – telophase, 6 – non-mitotic (negative), and 7 – atypical mitotic figures. In the LUNG-MITO dataset^24^, the number of annotations per category is: 799 (Category 1), 807 (Category 2), 1200 (Category 3), 103 (Category 4), 298 (Category 5), 620 (Category 6), and 1596 (Category 7). In the MIDOG++ dataset, the corresponding counts are: 2522 (Category 1), 2458 (Category 2), 4112 (Category 3), 327 (Category 4), 417 (Category 5), 14347 (Category 6), and 2100 (Category 7). In the COCO annotation files, segmentation masks are encoded as lists of polygon points in the format [x0, y0, x1, y1,…, xn, yn], and bounding boxes are specified using the [x_min, y_min, width, height] format. The source code is available at: (https://github.com/biomag-lab/Mitosis-detection).

## Technical Validation

The datasets were partitioned as follows: a subset of stMIDOG++ was used exclusively for training, while the LUNG-MITO dataset^24^ was reserved for evaluation to assess the domain robustness of the algorithm. 70% of the stMIDOG++ dataset's^24^ images were allocated for training, 10% for validation (used for confidence threshold optimization and hyperparameter tuning), and 20% for testing. To enable fair evaluation, a 3-fold cross-validation was performed using the training and validation sets, with the test set kept fixed. All performance metrics were computed as the average across the three folds. To ensure an unbiased evaluation, domain leakage between training, validation, and test sets was avoided by preventing overlap of crops at the patient level. Furthermore, the inclusion of the LUNG-MITO dataset^24^ ensures that domain robustness is rigorously assessed.

### Model performance

To benchmark the performance of the algorithm, comparisons were made against a classical Mask R-CNN architecture with a ResNet-50 backbone. Additionally, the contribution of the refinement step was assessed by evaluating the ConvNeXt-based method both with and without the refinement component.

All three algorithms were trained with the same hyperparameters and on the same folds. All three algorithms were based on the Mask R-CNN implementation of the MMDetection library^39^.

**Fig. 4:**
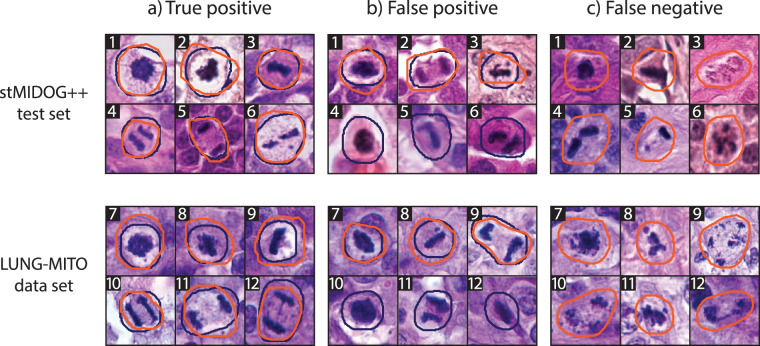
Representative examples of model performance on the stMIDOG++ test set and the LUNG-MITO data set. Ground truth (GT) color: orange. Predicted (P) color: deep blue. Magnification: 40×. Column (**a**) True positive detections from all normal phases and atypical mitosis. Column (**b**) False positive detections b1: GT: prometaphase, P: prophase. b2: GT: telophase, P: anaphase. b3: GT: atypical (metaphase), P: metaphase. b4: GT: none, P: prophase. b5: GT: none, P: metaphase. b6: GT: none, P: anaphase. b7: GT: prometaphase, P: prophase. b8: GT: atypical (metaphase), P: metaphase. b9: GT: atypical (telophase), P: telophase. b10: GT: none, P: prophase. b11: GT: none, P: anaphase. b12: GT: none, P: metaphase. These examples highlight characteristic misclassification modes, including confusion between adjacent mitotic stages and misclassification of non-mitotic cells as mitotic figures. Column (**c**) False negative detections c1: GT: prophase, P: none. c2: GT: metaphase, P: none. c3-c12: GT: atypical, P: none. These examples illustrate typical failure modes, including sensitivity to atypical morphology, and early mitotic stages.

Our results (Fig. 4) show that incorporating a ConvNeXt backbone can significantly enhance the performance of Mask R-CNN both on the stMIDOG++ and the LUNG-MITO datasets^24^. As for the refinement part, we can see that a significant part of the additional performance is yielded by this post-processing step (Table 1).

**Table 1:** Comparison of detection performance across models.

stMIDOG++ test set						
Model	F1	Precision	Recall	cw-F1	cw-Precision	cw-Recall
Mask R-CNN (ResNet-50)	0.683	0.641	0.731	0.485	0.455	0.519
Ours	0.767	0.768	0.766	0.585	0.586	0.584
Ours (refinement)	0.794	0.774	0.818	0.6	0.584	0.617

LUNG-MITO						
Model	F1	Precision	Recall	cw-F1	cw-Precision	cw-Recall
Mask R-CNN (ResNet-50)	0.570	0.658	0.503	0.334	0.386	0.295
Ours	0.691	0.798	0.611	0.462	0.534	0.408
Ours (refinement)	0.730	0.779	0.687	0.596	0.637	0.561

Metrics are reported as average values over three cross-validation folds (*cw* stands for class-wise score).

## Supplementary information

Supplementary Information

## Data Availability

The subtyping annotations for MIDOG++ and the LUNG-MITO images with annotations are available at 10.5281/zenodo.18661323.

## Code availability

Source code for model configuration, training, prediction and evaluation is available at https://github.com/biomag-lab/Mitosis-detection.
