# Supplementary material for: A Subphase-Labeled Mitotic Dataset for AI-powered Cell Division Analysis
Source: Sci Data. 2026 Mar 13;13:680. doi: 10.1038/s41597-026-07007-7 (PMC13128913; doi:10.1038/s41597-026-07007-7)
Supplement: Supplementary file 1 — Supplementary Information [file 41597_2026_7007_MOESM1_ESM.pdf]

Table of contents

Figure S1 2

Table S1 3

Table S2 5

Figure S1

Annotation strategy with example mitotic and mitotic-like figures

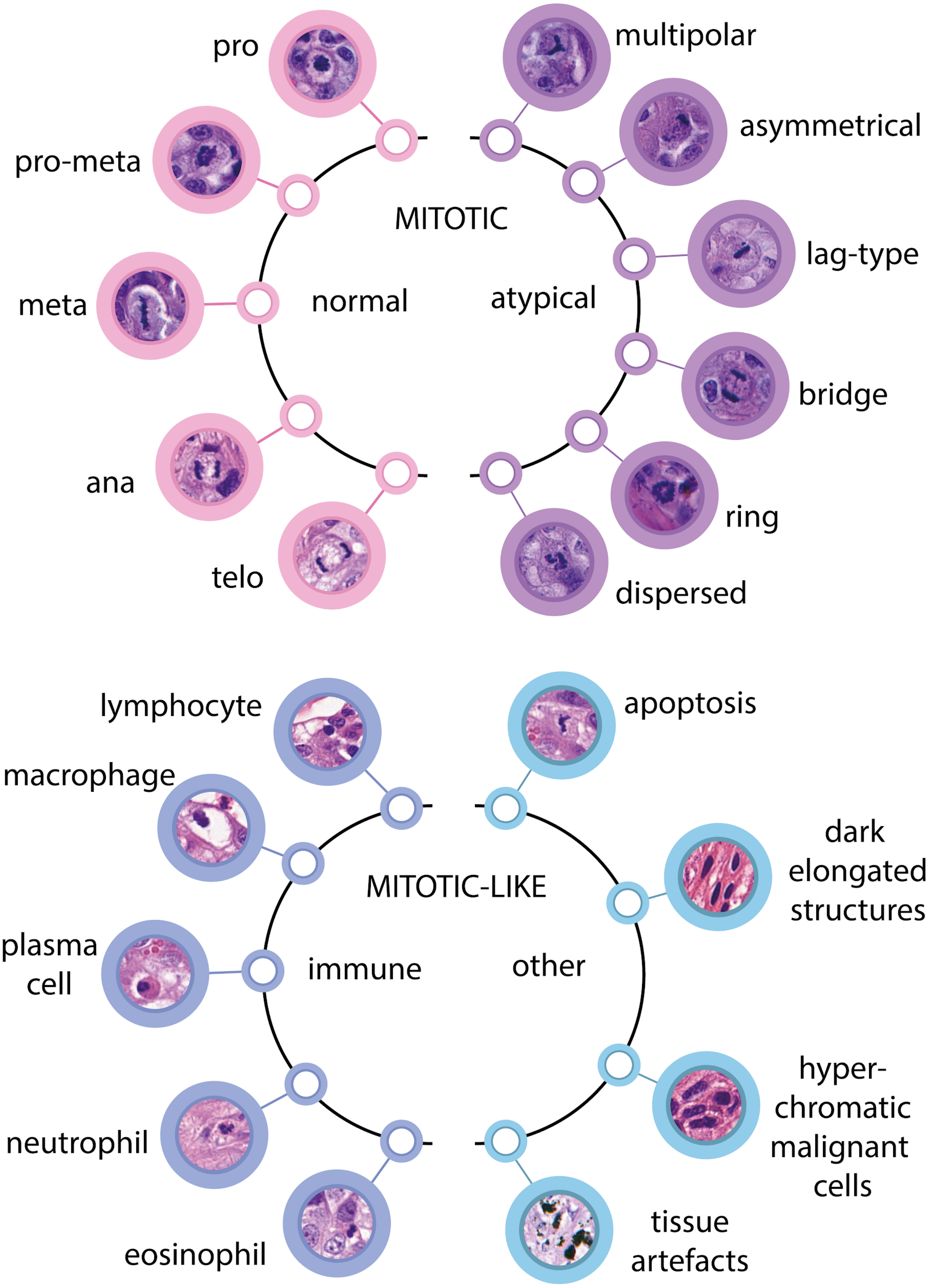

Table S1

Detailed sample processing protocol

Sample preparation step	Reagent	Time	Note
**Tissue fixation**	10% Formaldehyde	Overnight	Room temperature
**Paraffination**	70% v/v Ethanol in water	2x30 minutes	
	90% v/v Ethanol in water	30 minutes	
	96% v/v Ethanol in water	30 minutes	
	2:1 propan-2-ol and 96% Ethanol in water	3x1.5 hours	
	1:1 propan-2-ol+Xylene	30 minutes	
	Xylene	2x1 hour	
	Paraffin	4x1 hour	The sample stays in the last paraffin till the morning
**Tissue embedding**	Paraffin	30 minutes	Paraffin block generation
**Specimen slicing with microtome**	Water bath 46°C	10 minutes	Thickness: 5µm
**H&E staining**	Xylene	2 minutes	
	Xylene	2 minutes	
	Absolute Ethanol	1 minute	
	70% Ethanol in water	1 minute	
	50% Ethanol in water	1 minute	
	Distilled water	1 minute	
	Hematoxylin	1 minute	
	Tap water	2,5 minutes	
	Tap water	2,5 minutes	

	Eosin Y	30 seconds	
	70% Ethanol in water	1 minute	
	Absolute Ethanol	1 minute	
	Absolute Ethanol	1 minute	
	Xylene	2 minutes	
	Xylene	2 minutes	
**Coverslipping with mounting medium**	Leica Surgipath Micromount	10 minutes	50 µl/sample<br>Size of coverslip: 22x40 mm<br>Let it cure for 20 minutes before imaging

Table S2

Morphological characteristics of mitotic cells and mitotic-like figures

			Label	Cytoplasm	Chromosomes	Nuclear membrane	Nucleolus	Size, shape	Other
Mitosis	Normal	Normal mitosis	Prophase	abundant, eosinophillic, granular	condensed, uniform appearance as dark aggregates	nuclei with retained rounded contour, intact membrane	gradually disappears	cell shape: round or oval	
			Prometaphase		dark cluster with protruding rods and spikes or rosette (clear space within the central nuclear aggregate)	disintegrates	absent	cell shape: round	
			Metaphase		dark cluster with rods and spikes (linear plate, band or ring), aligned along the metaphase plate at the cell's equatorial plane.	absent	absent	cell shape: typically round with a clear equatorial plate	spindles: sometimes can be seen radiating outward from the chromosomes
			Anaphase		two (equally) separated aggregates (rods) with spikes or projections with variable distance (continuum)	absent	absent	cell shape: becomes more oval-shaped, elongated	sister chromatids may take a V shape
			Telophase		two separated aggregates with spikes or projections, at the opposite ends of the cell, begin to uncoil	reforms around each set of chromosomes, not always seen	within the newly formed nuclei, not always seen	cell shape: elongated, maximum distance of cells: 1 tumor cell	cleavage furrow: present. Cytokinesis: often begins, sometimes a thin strand of connecting cytoplasm is not visible
	Atypical	Polar asymmetry	Multipolar mitosis		presence of more than two spindle poles, leading to unequal chromosome segregation, hairy outlines.	absent	absent	size: enlarged	
			Asymmetrical mitosis		unequal cluster sizes (can be a sectioning artefact as well)	absent	absent	size: enlarged	
		Abnormal chromosome segregation	Lag-type mitosis		condensed chromatin not attached to a bigger cluster in the area of the mitotic figure. Can appear as chromosomes lagging between two separating masses of chromosomes during anaphase, at one side of the metaphase plate or both sides of the metaphase plate	absent	absent		
			Chromatin bridge		chromatin strands connecting daughter nuclei, suggesting incomplete chromosomal separation	absent	absent		
			Ring mitosis		displaced to the periphery of the cell, should be differentiated from ring metaphase	absent	absent		
			Dispersed mitosis		scattered chromosomal material without clear alignment, dispersed, non-clumped	absent	absent	size: enlarged	
Mitotic-like figures		Cell death	Apoptotic cells	dense, eosinophillic (red, pink), can be retractile	homogenous mass of round, basophillic degraded DNA with smooth outer contour, no distinct chromosome. Nuclei: can be pyknotic (small, dark). Karyorrhexis: irregular, fragmented nuclear material, intensely basophilic, but lacks the organized structure of mitotic chromosomes<br>Karyolysis: dissolution of the nucleus with faded or absent staining, not observed in true mitotic figures	Often remains intact during early stages	usually not visible	size: smaller, cellular membrane: detaches at the end. If it is close to necrotic area, more likely MLF (if close to hot spot and intact tissue, more likely MF)	
		Immune cells	Lymhocytes	scant, barely visible	stained, lacking the granular texture seen in mitotic chromatin	present	usually not visible	cell size and shape: small, round cells with low amount of cytoplasm	
			Foamy macrophages	abundant, vacoulated	dark, oval or indented	present	usually not visible	size: large,	
			Plasma cells	abundant, basophillic	eccentric	present	usually not visible	contains a pale zone of the Golgi apparatus. Shape: ovoid	
			Neutrophils	pale, pink	single, multilobed nucleus, usually segmented into 2 to 5 lobes connected by thin strands of chromatin	present	usually not visible	shape: round when inactive; amoeboid when activated	
			Eosinophils	abundant with eosinophillic granules	pycnotic, lobulated	present	usually not visible	shape: round	
		Hyperchromatic structures	Dark elongated structures	Compression or crushing of malignant cells with or without cytoplasm	smooth, regular outline, pycnotic, elongated, hyperchromatic	present	usually not visible	position: often at the edge of tumor clusters, compressed between cells, orientation: often aligned parallel to tissue structures	
			Hyperchromatic malignant cells	scant to moderate, color dependent on cell type	hyperchromasia of malignant cells (can be mistaken to prophase), smooth outline	present	can be prominent or not visible		
		Tissue artefacts						Formalin pigments: black to brown finely granular birefringent deposit<br>Delayed fixation: cell shrinkage, cytoplasmic clustering<br>Haemosiderin deposition	
